# Real-World Data in Support of Short Sleep Duration with Poor Glycemic Control, in People with Type 2 Diabetes Mellitus

**DOI:** 10.1155/2019/6297162

**Published:** 2019-05-21

**Authors:** Wagner Martorina, Almir Tavares

**Affiliations:** Postgraduate Program in Neurosciences, Federal University of Minas Gerais, Belo Horizonte, Brazil

## Abstract

**Aims:**

Sleep duration (SD) has been associated with metabolic outcomes. Is there an independent association between short/long SD and glycemic control (GC) in type 2 diabetes mellitus (T2DM) outpatients, compared to intermediate SD? Employing up-to-date definitions of SD, we comprehensively considered, simultaneously, all known confounding/mediating factors that recently emerged in the literature: age, gender, diet, physical activity, obesity, night pain, nocturnal diuresis, sleep quality, chronotype, sleep apnea, depressive symptoms, alcohol, caffeine, tobacco, number of endocrinologist appointments, T2DM family history, and sleep medication.

**Methods:**

A cross-sectional study of 140 consecutive T2DM outpatients, ages 40-65, *glycohemoglobin* (*HbA*_1*c*_) *goal* ≤ 7. We searched for variables (including HbA_1c_) significantly associated with short (<6 hours) or long (>8 hours) SD, in comparison to intermediate SD (6-8 hours).

**Results:**

Higher HbA_1c_ levels increased the chance of belonging to the group that sleeps <6 hours (*p* ≤ 0.001). Better sleep quality, nocturnal diuresis, and morningness increased the chance of belonging to the group that sleeps >8 hours (*p* < 0.05).

**Conclusions:**

There is an independent association between short SD and elevated HbA_1c_, in real-world T2DM outpatients. Future interventional studies could evaluate weather consistent, long-term sleep extension, from <6 hours to 7–9 hours per 24 hours, improves GC in T2DM outpatients.

## 1. Introduction

451 million people worldwide had diabetes mellitus in 2017, according to the International Diabetes Federation [1]. By 2045, numbers will increase to 693 million [1]. Recent data in Brazil (2014) estimates a prevalence of 19.7% in the general population [2], which is second only to China, India, and the USA [2]. Around 90% are cases of type 2 diabetes mellitus (T2DM). These estimates point to staggering increases in costs associated with the complications of this progressive disease: extremity amputations, end-stage renal disease, and cardio- and cerebrovascular disease [3]. The prevention of the emergence of the disease and the promotion of its adequate control are the only ways to reduce these impacts.

Since the Epic of Gilgamesh (second millennium BC), the ideal sleep duration (SD) and its deviations are a matter of medical debate [4]. The National Sleep Foundation (NSF) regularly reviews recommendations for SD, updating them according to the most current research. Presently, the NSF recommends 7 to 9 hours of sleep per day for the group with ages between 26 and 64 years old, safeguarding, however, that 6 to 10 hours may be appropriate [5]. Such directions originated from specialists' consensus and were supported by a review of 312 articles published between 2004 and 2014 [5]. SD is amenable to behavioral recommendations and interventions, inexpensive in biological terms.

Recent (2018) epidemiological studies suggest that both short and long SD are associated with an increased risk of acquiring T2DM, in comparison to intermediate SD [6, 7]. A small group of studies [8–13] analyzed whether short/long SD is deleterious to glycemic metabolism, in patients already diagnosed with T2DM. In some of these studies, short/long SD is associated with worse glycemic control (GC), assessed by glycohemoglobin (HbA_1c_) levels [8–11].

The investigation of the association between SD and GC in T2DM patients is a challenging enterprise. Recent literature indicates that multiple confounding variables are to be considered, particularly obesity [6], obstructive sleep apnea (OSA) [14], chronotype [15], sleep quality [16], and depressive symptoms [17]. In addition to that, in order to evaluate whether this association occurs independently, known mediating factors must be considered: diet [18], physical activity [19], nocturnal diureses [20], pain nocturnal [20], and alcohol use [21]. Finally, it is necessary to take into account that the relationship between SD and GC is bidirectional: SD interferes with GC [22], and hyperglycemia symptoms (particularly, nocturia and diabetic neuropathy) interfere with SD [20]. To the best of our knowledge, previous studies were not able to include all these factors simultaneously.

The pathophysiology of the sleep-T2DM association is not completely understood, and several hypotheses are to be considered. Experimental studies in healthy humans point that short SD can lead to glucose intolerance [23], decreased insulin sensitivity [24], and worsening of food intake quality [25]. Similar mechanisms were proposed to explain a relationship found between glycemic metabolism and long SD [26].

The aim of this study is to determine the association between SD and GC in real-world outpatients already diagnosed with T2DM persists, when all confounding and mediating variables that emerged in recent sleep-T2DM literature are simultaneously included in the study analysis.

## 2. Methods

### 2.1. Ethical Aspects

This work was approved by the Research Ethics Committee of the Federal University of Minas Gerais (protocol CAE: 63951317.5.0000.5149). All participants signed an informed consent form for this study, conforming to the standards of the Helsinki Declaration.

### 2.2. Design

Clinical data collection for this cross-sectional study took place from April to September 2017. A total of 140 patients diagnosed with T2DM, aged 40 to 65 years, from a health network of the metropolitan region of Belo Horizonte, Brazil, were included. The exclusion criteria were as follows: (1) age < 40 or >65; (2) pregnancy; (3) recent (<3 months) corticosteroid use; (4) recent (<1 year) diagnosis of T2DM; and (5) conditions that would render glycemic target (HbA_1c_ < 7%) more flexible (end-stage renal disease, recent acute coronary syndrome, and other major illnesses, such as cancer and hepatic insufficiency).

### 2.3. Clinical and Laboratory Evaluation

All participants were interviewed and examined by the same board-certified endocrinologist (WM). SD was evaluated with the question “How long do you sleep in a 24-hour period?” This encompassed both night- and daytime sleep segments. Previous studies on the association between SD and CG similarly analyzed sleep in a 24-hour period [8, 11]. Such methodology is supported by a work suggesting that diurnal naps can reduce the negative impact of sleep restriction in GC [5, 11]. Participants were divided into three categories according to SD: short sleep (<6 hours/24 hours), intermediate sleep (6-8 hours/24 hours), and long sleep (>8 hours/24 hours). The risk of OSA, a potential confounder of the relationship between SD and GC, was determined by the Brazilian version of the STOP-BANG questionnaire [27] (score ≥ 3 used as a risk indicator). Excessive daytime somnolence (EDS) was evaluated by the Brazilian version of the Epworth Sleepiness Scale [28] (score > 10 identified EDS). The Brazilian version of Stunkard and Messick's [29] Three Factor Eating Questionnaire R-21 [30] was utilized to assess three domains: (a) cognitive restraint of eating (eating behaviors involving obligations and prohibitions); (b) uncontrolled eating (as a result of a loss of control over the intake); and (c) emotional eating (in response to negative emotional states). To evaluate depressive symptoms, the Patient Health Questionnaire (PHQ-9) was utilized [31]. Because the study population was not composed of young students and consisted mostly in middle-aged patients, we separated Horne and Östberg's Morningness-Eveningness Questionnaire scores according to cut-off points recommended by Taillard et al. [32] (morning type ≥ 65; neither type 53 to 64; evening type ≤ 52), specifically determined to classify chronotypes in a population of middle-age adults. Sleep quality was assessed according to the modified Pittsburgh Sleep Quality Index (mPSQI), proposed by Knutson et al. [9], which evaluates sleep quality independently of SD. Weekly frequencies of nocturnal pain (suggesting diabetic neuropathy) and of nocturia (suggesting poorer glycemic control) were counted, in order to estimate the influence of hyperglycemia symptoms on SD. The weekly frequency of alcohol ingestion, a parameter relevant for both GC and SD [21], was taken into account. Physical activity was established according to the minutes of physical exercises per week. Body mass index (BMI) was obtained by dividing weight in kilograms by squared height in centimeters. HbA_1c_ was evaluated by high performance liquid chromatography.

### 2.4. Statistical Analysis

Because the study data did not fit a normal distribution pattern, we tabulated the continuous variables according to medians and 25% and 75% quartiles (Table 1). For categorical variables, frequencies and proportions were listed.

Two generalized log-binomial linear models were adjusted to carry out the study. In the first model, short SD was compared to intermediate SD. In the second model, long SD was compared to intermediate SD. All variables with *p* value ≤ 0.20 were included in the multivariate model. Variables with higher values of *p* were removed stepwisely, and variables with *p* values ≤ 0.05 remained in the multivariate model (Table 2). Since the study design is cross-sectional, the prevalence ratio (PR) was used as a measure of association. The level of significance was set at 0.05. SPSS software version 20.0 was employed.

## 3. Results

140 consecutive patients were included, and Table 1 shows the distribution of the main variables for all participants. The median age was 56 (50-61) years. The majority were female (61.4%), worked (68.1%), slept 6 to 8 hours in a 24-hour period (69.5%), and presented with an increased risk for OSA (60.5%). Only 29.3% of the patients utilized insulin. The median for BMI was 30 kg/m^2^ (quartiles 25-75%: 26.9-33.5 kg/m^2^), and the median for HbA_1c_ was 7.3% (quartiles 25%-75%: 6.5%-8.7%).

Table 2 displays the variables that remained with *p* < 0.2 in a univariate comparison, for short SD versus intermediate SD (model S) and for long SD versus intermediate SD (model L). Model S shows that variables *time since TDM2 diagnosis*, *HbA_1c_*, and *PHQ-9 score* were significantly and positively associated with short SD, in comparison to intermediate SD (*p* < 0.05). Model L indicates that variables *education* and *modified PSQI* score were significantly and negatively associated with long SD, in comparison to intermediate SD. This model also showed that longer *time since DM2 diagnosis*, *insulin use*, higher *weekly frequency of nocturia*, and higher *STOP-BANG score* were significantly and positively associated with long SD (*p* < 0.05).

Table 3 displays the multivariate model for short SD in relation to intermediate SD. *HBA_1c_ level* was the only variable that remained in the model (PR 1.27, 95% CI: 1.12-1.44, *p* ≤ 0.001), demonstrating that an increase in its value increases the probability that such patients belong in the short SD group, in comparison to the intermediate SD group, independently of other variables.

Table 4 displays the multivariate model for long SD in relation to intermediate SD. An increase in the *MEQ score* independently increased the probability of belonging to the group with SD > 8 hours, in comparison with the group with SD between 6 and 8 hours (PR 1.05, 95% CI: 11.10, *p* = 0.004). Higher *frequency of nocturia throughout the week* was also significantly associated with long SD (PR 3.13, 95% CI: 1.25-7.84, *p* = 0.015). The model also showed that a reduction in the *modified PSQI score* (indicating better sleep quality) was independently associated with long SD (PR 0.74, 95% CI: 0.59-0.093; *p* = 0.009).

## 4. Discussion

The present study demonstrated that higher levels of HbA_1c_ are significantly and independently associated with a short SD (<6 hours in 24 hours), in comparison to an intermediate SD (6 to 8 hours in 24 hours), in a real-world population of Brazilian T2DM outpatients. This association remained significant after the simultaneous inclusion, in the analysis, of all of the most likely mediating and confounding factors that emerged in recent T2DM-sleep literature (Table 1): age, gender, blood pressure, OSA, time since T2DM diagnosis, sleep quality, chronotype, depressive symptoms, use of alcohol, obesity, physical activity, diet, nocturia, night pain, caffeine intake, smoking, number of endocrinologist appointments per year, family history of T2DM, and use of sleep-inducing medication. An association between long SD and HbA_1c_ was not identified.

Relevant methodological diversity is among the reasons why current publications discussing SD-GC in patients already diagnosed with T2DM apparently exhibit discrepant results.

Cooper et al. [12] and Williams et al. [13] did not find an association between SD and HbA_1c_. However, these two studies were primarily designed to evaluate cardiovascular risk in T2DM and were not designed for the evaluation of GC.

In comparison to intermediate SD, both short and long SD were associated with worse GC in patients already diagnosed with T2DM, in previous studies [8, 10, 11, 33]. The definition of short and long SD varied considerably in these studies, ranging from less than 4.5 to 7 hours for short SD and from 8 to 9 or more hours for long SD. For the present study, updated SD definitions were chosen, in conformity to current recommended standards [5], also employed in a recent systematic review and meta-analysis of SD and GC by Lee et al. [22]. In the present study, short SD was defined as <6 hours; intermediate SD, 6-8 hours; and, long SD, >8 hours. In the work of Lee et al. [22], both short and long SD were significantly related to higher HbA_1c_, when compared to intermediate SD. Lee et al. [22] point that several confounding factors of association could not be studied in their analysis, since most of the papers included in their work did not present such data. Due to the absence of this relevant information, under- or overestimation of the relationships between SD and GC may have occurred.

In 2013, Ohkuma et al. [8] found both short and long SD significantly associated with higher levels of HbA_1c_, when compared to intermediate SD (defined as 6.5 to 7.4 hours) (*p* for quadratic trend < 0.001). This work utilized self-reported SD, in a self-administered questionnaire: “How long is your usual sleep duration, including naps?”. In spite of the fact that the present study also employed a subjective question for the assessment of SD (“How long do you sleep in a 24-hour period?”), a fundamental difference is to be noted. The same board-certified endocrinologist (WM) asked that question to all patients in the present study, during an office-based, doctor-patient clinical interview, for the good of reliability, in an outpatient real-world environment. Unlike the study of Ohkuma et al. [8], a significant association between long SD and HbA_1c_ was not found in the present study, possibly due to several design differences between the studies. Firstly, the relationship between long SD and HbA_1c_ is attenuated in subjects aged <70 years [8], which is one of the samples of the present study, which included only patients with age ≤ 65. In addition, the sample of the present study consisted in 140 patients only and was much smaller to the sample of 4870 patients studied by Ohkuma et al. [8]. Nevertheless, it is relevant to point out that the present study took in consideration a most relevant confounding factor between GC and long SD: OSA is an indicator of intermittent hypoxemia. OSA is quite frequent among T2DM patients, with prevalences ranging from 58 to 86% [34]. Since the data of Ohkuma et al. [8] were not adjusted for OSA, a high prevalence of occult OSA in that study may have mediated the association between long SD and HbA_1c_. One of the risk factors for OSA is older age [35]. The mean age of patients in the study of Ohkuma et al. [8] (>65 years) is higher than the mean of age of patients in the present study.

In the studies of Gozashti et al. [11] and of Tang et al. [10], short SD was associated with poor GC and long SD with improved GC, in T2DM patients. The results of Gozashti et al. [11] and Tang et al. [10] were in the same direction to the results of the present study, in regard to short SD, and in the opposite direction in regard to long SD. OSA might be the reason for this discrepancy regarding long SD. In studies of Gozashti et al. [11] and Tang et al. [10], OSA participants were excluded when they self-reported such condition. This might suggest that OSA did not interfere with the relation between long SD and poor GC in these two studies. However, OSA is frequently underdiagnosed in the diabetic population [36], and a considerable number of OSA cases may have remained undetected in the populations of Gozashti et al. [11] and Tang et al. [10]. Because OSA is such a relevant phenomenon for the long-term care of complications of T2DM [37], we avoided excluding OSA patients, and we opted to measure the *OSA risk*, in pursuit of a real-life clinical relevance. The STOP-BANG questionnaire [38], a validated method to evaluate the OSA risk, was chosen. The use of this improved method to appraise the influence of the *OSA risk* as a confounding factor may have determined the absence of association between long SD and HbA_1c_ in the present study, in contrast to the studies of Gozashti et al. [11] and Tang et al. [10]. In a study of 161 T2DM that excluded cases with long SD, Knutson et al. [9] did not exclude OSA cases and analyzed the OSA risk. However, it is relevant to point that the OSA risk was measured by means of an unvalidated subjective instrument: weekly frequency of nocturnal snoring.

In the same line of search, Siwasaranond et al. [39] examined factors associated with poorer GC in T2DM patients with untreated sleep-disordered breathing (SDB), an umbrella term for chronic conditions with breathing cessations in sleep, most frequently OSA. They used objective methods to rate both SDB and SD, in contradistinction to subjective questioning: SDB was diagnosed when the apnea-hypopnea index (AHI) was ≥5, assessed by an overnight in-home somnographic monitoring device (WatchPAT 200), and SD was recorded by wrist actigraphy for 7 days. An association between OSA severity and GC in patients T2DM was suggested in a previous work [40], which was not confirmed by Siwasaranond et al. [39]. Only shorter SD was independently associated with higher HbA_1c_, in this study of Siwasaranond et al. [39].

The relevance of short SD as a mediator of worse GC in T2DM patients was also identified in a study that focused on sleep timing. Studying GC of T2DM patients in Thailand, Reutrakul et al. [33] showed that later bedtime on weekends was associated with poorer GC (*p* = 0.01). However, in a hierarchical logistic analysis, this association was totally mediated by shorter SD. Employing distinct methodologies, both Reutrakul et al. [33] and de Medeiros et al. [30] found a relevant association between short SD and GC, which was confirmed with a different study design in the present study.

In our first univariate analysis (Model S, Table 2), results were similar to those previously obtained by Ohkuma et al. [8]: a significant association between short SD and *depressive symptoms* (PHQ-9). Nevertheless, HbA_1c_ was the only variable associated with short SD, in comparison to intermediate SD. Therefore, the association between higher *HbA_1c_* level and short SD was not mediated by variables related to hyperglycemia symptoms, like *nocturia* and *nocturnal pain*. Confounding factors like *OSA*, *sleep quality*, *chronotype*, and *depressive symptoms* did not explain this association. Besides that, other factors commonly associated with poor GC in T2DM, like *sedentary lifestyle* and *inadequate diet*, were not significantly different in the studied groups. As the study design is cross-sectional, it is not possible to conclude that worse GC caused a shortening in SD, nor that short SD was the cause of worse GC. Therefore, an independent association between higher levels of HbA_1c_ and short SD was shown, without pointing to the cause-effect direction.

In the second univariate analysis (Model L, Table 2), long SD was negatively associated with *education* (years) and positively associated with *time since T2DM diagnosis*, *insulin use*, and *OSA risk* (STOP-BANG questionnaire score), in comparison with intermediate SD. *Education* median was high enough (11 years; Table 1) so as not to interfere with comprehension of the clinical interview, or with the use of questionnaires. Although *education* was significant in this univariate analysis (Model L, Table 2), it did not persist association with SD in the multivariate analysis. Similar associations between long SD and longer *duration of T2DM* and *insulin use* were also present in the study of Ohkuma et al. [8]. However, in the final analysis in the present study (multivariate, Model L, Table 2), only higher *MEQ score* (indicating morningness), higher number of *nocturia* days per week (indicating sleep interruptions), and lower *modified PSQI score* (indicating better quality of sleep) were independently associated with the probability of belonging to the group that slept >8 hours. The association found between long SD and better-quality sleep in the present study is contrary to the line of reasoning in the work of Ferrie et al. (The Whitehall II Study) [41] that long SD might be a mechanism to compensate for poor sleep quality due to OSA, depression, and other comorbidities. An increase in the number of *nocturnal diuresis* events was shown in our patients with long SD. Nonetheless, sleep quality was better in this group, in comparison to the group with intermediate SD. This may indicate that poor sleep quality at night caused by *nocturia* was compensated by some sort of sleep extension along the 24 hours of the day. This improved sleep quality in a 24-hour period could be an explanation for the absence of an inadequate GC in our patients with long SD.

In regard to pathophysiology, some experimental studies in healthy humans attempted to explain how sleep deprivation interferes with glycemic metabolism [23, 24, 42, 43]. Sleep restriction would lead to changes in satiety and hunger hormones (leptin and ghrelin) [25], reduction of physical activity [19], increased insulin resistance mediated by elevated cortisol, catecholamines [23], growth hormone, inflammatory cytokines [42], melatonin hormone, and the expression of genes related to circadian rhythm and glycemic metabolism [43]. In regard to *diet* and *physical activity*, the present study did not show significant differences when the short and long SD groups were compared to the intermediate SD group. The influence of insulin resistance was not evaluated in the present study.

The strength of the current work lies in the simultaneous inclusion in the analysis of the most recent and significant studied variables that could mediate or confuse the multifaceted relationship between SD and GC. The absence of these variables can be a problem in studies in this area, particularly the OSA risk [8, 22]. Although the study of Knutson et al. [9] analyzed the OSA risk, it was measured by means of an unvalidated instrument: weekly frequency of nocturnal snoring. In their studies, Gozashti et al. [11] and Tang et al. [10] only excluded patients that self-reported OSA and may have missed other OSA patients. In order to avoid that, in the present work, OSA was screened with the STOP-BANG questionnaire [27], an instrument validated for this purpose. The influence of *nocturia* and *night pain*, two very important mediating factors in the case of T2DM, was taken into account and evaluated. Previous studies excluded these factors only by self-report. In addition, chronotype, a variable previously related to SD and glycemic control, was included [15]. Finally, the prevalence ratio was used as a measure of association, which is more adequate to evaluate the association of disorders of high prevalence in a population [44]. Because the prevalence of sleep disorders in patients with T2DM is high, this choice is particularly relevant. Previous studies [8, 10, 11] used the odds ratio, a measure of association that overestimates the results when the prevalence of a characteristic in a population is high. In this aspect, therefore, the results of the present study can be considered more robust, and this may have contributed to the lack of association found between long SD and GC.

The main limitations of the current work regard the lack of utilization of objective measurements (polysomnography to measure SD and to evaluate OSA and actigraphy to estimate SD). The OSA risk was evaluated with a subjective instrument (the STOP-BANG questionnaire), and SD was obtained in a subjective clinical interview. The subjective nature of these data may interfere with the reliability of the association between SD and GC. However, the methodology was based on previous studies indicating that SD measured by actigraphy or polysomnography correlates reasonably well with self-reported SD [45]. The risk of OSA was defined by a STOP-BANG *score* ≥ 3 (intermediate risk). Given the high sensitivity of this method [46], it is unlikely that patients with this disorder were not categorized in the at-risk group. In addition, the study had a cross-sectional design, and thus, causality and directionality between SD and GC cannot be inferred. Nonetheless, potential mechanisms that could influence the SD and CG of outpatients with T2DM were considered and none of them mediated this relationship.

## 5. Conclusion

This work demonstrated an association between SD < 6 hours per day and worse glycemic control in real-world T2DM outpatients. This association did not result from confounding or mediating factors that recently emerged in the sleep-diabetes scientific literature. The relationship between SD and GC is worth interventional studies, with consistent, long-term sleep extension, from <6 hours to 7–9 hours per 24 hours, to evaluate weather this modulates improvements in GC.

## Figures and Tables

**Table 1:** 140 patients with type 2 diabetes mellitus.

Variables	*N* (%) or median (quartile 25% and 75%)
Age (years)	56 (50-61)
Female gender	86 (61.4)
Caucasian	68 (48.2)
Education (years)	11 (7-15)
Employed	96 (68.1)
Time since diagnosis of T2DM	7 (3-10)
Family history of T2DM	104 (74.3)
Endocrinologist visits/year	3 (2-4)
Physical activity (minutes/week)	0.0 (0-180)
% uncontrolled eating (TFEQ-21)	22.2 (11.11–47.0)
% emotional eating (TFEQ-21)	16.6 (0–4.4)
% cognitive restraint eating (TFEQ-21)	44.4 (7.7-61.1)
Number of insulin users	41 (29.3)
HbA_1c_ (%)	7.3 (6.5-8.7)
BMI (kg/m^2^)	30 (26.9-33.5)
Systolic blood pressure	120 (120-140)
Diastolic blood pressure	80 (80-80)
Number of patients with nocturnal pain days per week ≥ 1	62 (44.3)
Nocturia days/week	2 (1-3)
Number of patients that used alcohol > 1 time/week	51 (36.4)
Number of smokers	13 (9.3)
Caffeine intake (mg/day)	190 (95-295)
Pittsburgh Sleep Quality Index (modified)	7 (5-10)
Epworth Sleepiness Scale score	9 (6-12)
Number of patients with Epworth score > 10	50 (35.7)
Chronotype (Morningness-Eveningness Questionnaire) score	64 (59-69)
Chronotype:	
Morningness	66 (47.7)
Intermediate	58 (41.4)
Eveningness	16 (11.4)
STOP-BANG Questionnaire score	3 (2-4)
Number of patients with STOP-BANG Questionnaire score ≥ 3	85 (60.7)
PHQ-9 score	9 (5-14)

*N*: number; T2DM: type 2 diabetes mellitus; HbA_1c_: glycohemoglobin; BMI: body mass index; PHQ-9: Patient Health Questionnaire.

**Table 2:** Prevalence ratios for short and long sleep durations, compared to intermediate sleep duration. Variables with *p* ≤ 0.2 in the univariate log-binomial analysis.

Variables	Model S: short sleep duration	Model L: long sleep duration
	PR (95% CI)	PR (95% CI)
Female gender	1.53 (0.83–2.81)^∗^	1.50 (0.49–4.59)
Education (years)	0.95 (0.89–1.02)^∗^	0.86 (0.76-0.98)^∗∗^
Time since T2DM diagnosis (years)	1.06 (1.01–1.10)^∗∗^	1.12 (1.02–1.22)^∗∗^
Number of insulin users	1.33 (0.70–2.53)	4.40 (1.39–13.99)^∗∗^
Number of patients that used alcohol > 1 time per week	1.75 (0.96–3.20)^∗^	2.43 (0.80–7.44)^∗^
HbA_1c_ (%)	1.28 (1.13–1.44)^∗∗^	1.25 (0.94–1.66)^∗^
Caffeine intake (mg/day)	1.001 (0.999–1.002)	1.001 (1.001–1.003)^∗^
STOP-BANG Questionnaire score	1.11 (0.96–1.29)^∗^	1.31 (1.03-1.68)^∗∗^
Number of patients with STOP-BANG Questionnaire score ≥ 3	1.51 (0.78–2.95)	7.03 (0.93-53)^∗^
Chronotype (MEQ score)	1.01 (0.98–1.03)	1.04 (0.99–1.10)^∗^
Cognitive restriction (%)	0.987 (0.97–1.00)^∗^	0.98 (0.96–1.02)
Emotional eating (%)	1.01 (0.99–1.020)^∗^	0.98 (0.96–1.0)^∗^
PHQ-9 score	1.06 (1.02–1.10)^∗∗^	0.96 (0.85–1.07)
Nocturia days per week	0.98 (0.75–1.29)	3.47 (1.28–9.42)^∗∗^
Number of patients with night pain days per week ≥ 1	1.80 (0.96–3.36)^∗^	1.77 (0.57-5.45)
Modified Pittsburgh Sleep Quality Index	1.03 (0.94–1.13)	0.80 (0.67–0.95)^∗∗^

PR: prevalence ratio; 95% CI: 95% confidence interval; T2DM: type 2 diabetes mellitus; HbA_1c_: glycohemoglobin; MEQ: Morningness-Eveningness Questionnaire; PHQ-9: Patient Health Questionnaire; ^∗^0.2 ≥ *p* > 0.05; ^∗∗^*p* < 0.05.

**Table 3:** Multivariate log-binomial model for short sleep duration.

Independent variable	Dependent variable: short SD compared to intermediate SD		
	Prevalence ratio	95% CI	*p* value
HbA_1c_	1.27	1.12–1.44	*p* ≤ 0.001

SD: sleep duration; 95% CI: 95% confidence interval; HbA_1c_: glycohemoglobin.

**Table 4:** Multivariate log-binomial model for long sleep duration.

Independent variables	Dependent variable: short SD compared to intermediate SD		
	Prevalence ratio	95% CI	*p* value
MEQ score	1.05	1.00–1.10	0.040
Nocturia (days/week)	3.13	1.25–7.84	0.015
mPSQI	0.74	0.59–0.93	0.009

SD: sleep duration; 95% CI: 95% confidence interval; MEQ: Morningness-Eveningness Questionnaire; mPSQI: modified Pittsburgh Sleep Quality Index.

## Data Availability

The database used to support the findings of this study are available from the corresponding author upon request.
